# A revised terminology for the pharyngeal arches and the arch arteries

**DOI:** 10.1111/joa.13890

**Published:** 2023-05-29

**Authors:** Anthony Graham, Jill P. J. M. Hikspoors, Robert H. Anderson, Wouter H. Lamers, Simon D. Bamforth

**Affiliations:** ^1^ Centre for Developmental Neurobiology, King's College London London UK; ^2^ Department of Anatomy & Embryology Maastricht University Maastricht ER The Netherlands; ^3^ Biosciences Institute, Faculty of Medical Sciences Newcastle University, Centre for Life Newcastle UK

**Keywords:** arch arteries, pharyngeal arches

## Abstract

The pharyngeal arches are a series of bulges found on the lateral surface of the head of vertebrate embryos. In humans, and other amniotes, there are five pharyngeal arches and traditionally these have been labelled from cranial to caudal—1, 2, 3, 4 and 6. This numbering is odd—there is no ‘5’. Two reasons have been given for this. One is that during development, a ‘fifth’ arch forms transiently but is not fully realised. The second is that this numbering fits with the evolutionary history of the pharyngeal arches. Recent studies, however, have shown that neither of these justifications have basis. The traditional labelling is problematic as it causes confusion to those trying to understand the development of the pharyngeal arches. In particular, it creates difficulties in the field of congenital cardiac malformations, where it is common to find congenital cardiac lesions interpreted on the basis of persistence of the postulated arteries of the fifth arch. To resolve these problems and to take account of the recent studies that have clarified pharyngeal arch development, we propose a new terminology for the pharyngeal arches. In this revised scheme, the pharyngeal arches are to be labelled as follows—the first, most cranial, the mandibular (M), the second, the hyoid (H), the third, the carotid (C), the fourth, the aortic (A) and the last, most caudal, the pulmonary (P).

## Graphical abstract

Three‐dimensional (3D) reconstructions of the developing pharyngeal region of the human embryo between 29 and 34 days post‐fertilisation (CS11–CS14), revealing the appearance of the pharyngeal pouches, along with the arteries and nerves that extend through the arches delimited by the pouches.

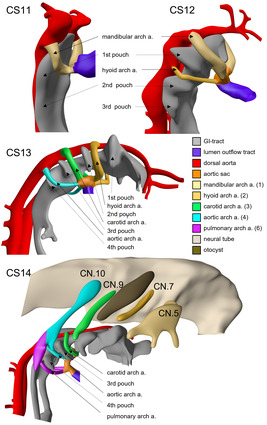

## 1 INTRODUCTION

Our aim in this review is to offer a reconsideration of the significance and the terminology of the pharyngeal arches. This is a critical issue to address, as it has importance for basic scientists, clinicians and the many students: biological, medical and dental, for whom an understanding of the pharyngeal arches is important. The current description of the pharyngeal arches and their import, which is found in the standard, and widely read, textbooks of embryology and anatomy (e.g.—see [Schoenwolf, 2014, Standring, 2015]) and which is spread across the Internet, with numerous videos, is erroneous. This situation has arisen from a mishmash of evolutionary, embryological and anatomical considerations, which have been in place since the early twentieth century, but which do not stand up to scrutiny.

The pharyngeal arches are bilateral structures conjoined at their ventral midline. In humans, and other amniotes, there are five arches, currently numbered from cranial to caudal, 1, 2, 3, 4 and 6. They consist of epithelial layers, ectoderm externally and endoderm internally, which surround neural crest cells. The neural crest cells, in turn, surround a mesodermal population. These different embryonic populations will differentiate to form a range of tissues. The neural crest‐derived cells give rise to skeletal and connective tissues, the mesoderm to muscle and endothelium, the ectoderm to the skin and sensory neurons, and the endoderm to the lining of the pharynx and specialised organs (Graham & Richardson, 2012; Schoenwolf, 2014).

## 2 EVOLUTIONARY CONSIDERATIONS

The variations between different vertebrate groups underpin many discussions about the pharyngeal arches and their significance, including in humans. There are good reasons for this. The pharyngeal arches are a series of bulges found on the lateral surface of the head of all vertebrate embryos (Graham & Richardson, 2012). Indeed, their presence is a defining feature of the vertebrate phylotypic stage. This is the period during which embryos from different vertebrate classes are most closely related in terms of morphology and gene expression (Irie & Kuratani, 2011). In many vertebrate clades, the embryonic segmental series of the pharyngeal arches is translated into the mature anatomy. This underpins the serial arrangement of the nerves, skeletal elements and the muscles and arteries of the mouth and pharynx.

Looking across the vertebrates, it is apparent that there are different numbers of pharyngeal arches in different clades. The basal condition for jawed vertebrates is to have seven pharyngeal arches. These will give rise to the jaw, the hyoid and five gill arches. Within the lineage leading to the amniotes, the sarcopterygians, there has been a trend towards a reduction in the number of pharyngeal arches (Shone et al., 2016). The coelacanth *Latimeria* displays the ancestral state and has seven pharyngeal arches, while amphibia have six arches and amniotes five arches. This reduction in the number of arches has accompanied the assumption of a terrestrial lifestyle. This is fully realised in the amniotes, which have the lowest number of arches. The analysis of HOX gene expression patterns in the developing pharyngeal region across different vertebrates has shown that this reduction in the number has involved the loss of post‐otic pharyngeal segments (Graham et al., 2019; Shone et al., 2016).

It has also long been thought that the relationship between the embryonic segmental units and the adult anatomy, as is seen in anamniotes, also pertain to humans, and other amniotes. A common feature of anatomy and embryology textbooks is the presence of a table that directly relates each of the pharyngeal arches with the components of the later jaw and pharyngeal and laryngeal anatomy. In amniotes, however, once the full complement of five arches has been generated, they undergo significant remodelling. As a result, in this clade, the caudal pharyngeal arches are transient and during their limited time of existence, they do not generate distinct muscular and skeletal elements (Poopalasundaram et al., 2019). The muscle and skeletal components of the larynx are formed at later stages after the caudal pharyngeal arches have been remodelled (de Bakker et al., 2017; Poopalasundaram et al., 2019). Thus, while distinct muscular and skeletal elements can be ascribed to the cranial arches, this is not so for the caudal arches.

## 3 NUMBERING OF THE ARCHES

This issue is sometimes tied to the evolutionary considerations. In amniotes, including humans, as we have already indicated, the first four are numbered sequentially. The ultimate arch, however, is numbered as being the sixth. This is odd and confusing. Two reasons are given for the missing fifth arch. One has been that the ‘fifth’ arch is transient, forming but never being realised. As we will discuss further, there is no evidence to support this assertion. A second reason given is that, by labelling the last arch as being the sixth, affinity is made between this final arch as seen in amniotes with the terminal arch of the other tetrapod clade, the amphibia, which have six pharyngeal arches (discussed in ‐ Graham et al., 2019). This hypothesis has been tested and does not stand up to scrutiny. In all vertebrates, the caudal limit of the pharyngeal arches is homologous (Shone et al., 2016). It is marked by the expression of HOX1 genes in the most caudal pharyngeal pouch, it is skirted by the hypoglossal (XIIth) nerve and is the site of origin of the ultimobranchial body. We could argue that the numbering of the final arch in amniotes should reflect its affinity with the last arch of the larger clade to which tetrapods belong, namely the osteichthyans. This clade has seven arches. So, in amniotes, why not label the arches as one through four, and then seven? These previous arguments mean that two points need to be addressed. The first is the direct relationships, if any, between the pharyngeal arches and the later anatomy. The second is their numbering. These issues are connected. The problems are resolved by considering the early development of the pharyngeal arches. This then permits the correlations to be made between the embryonic organisation and the later anatomy.

## 4 THE DEVELOPMENT OF THE PHARYNGEAL ARCHES

**FIGURE 1:**
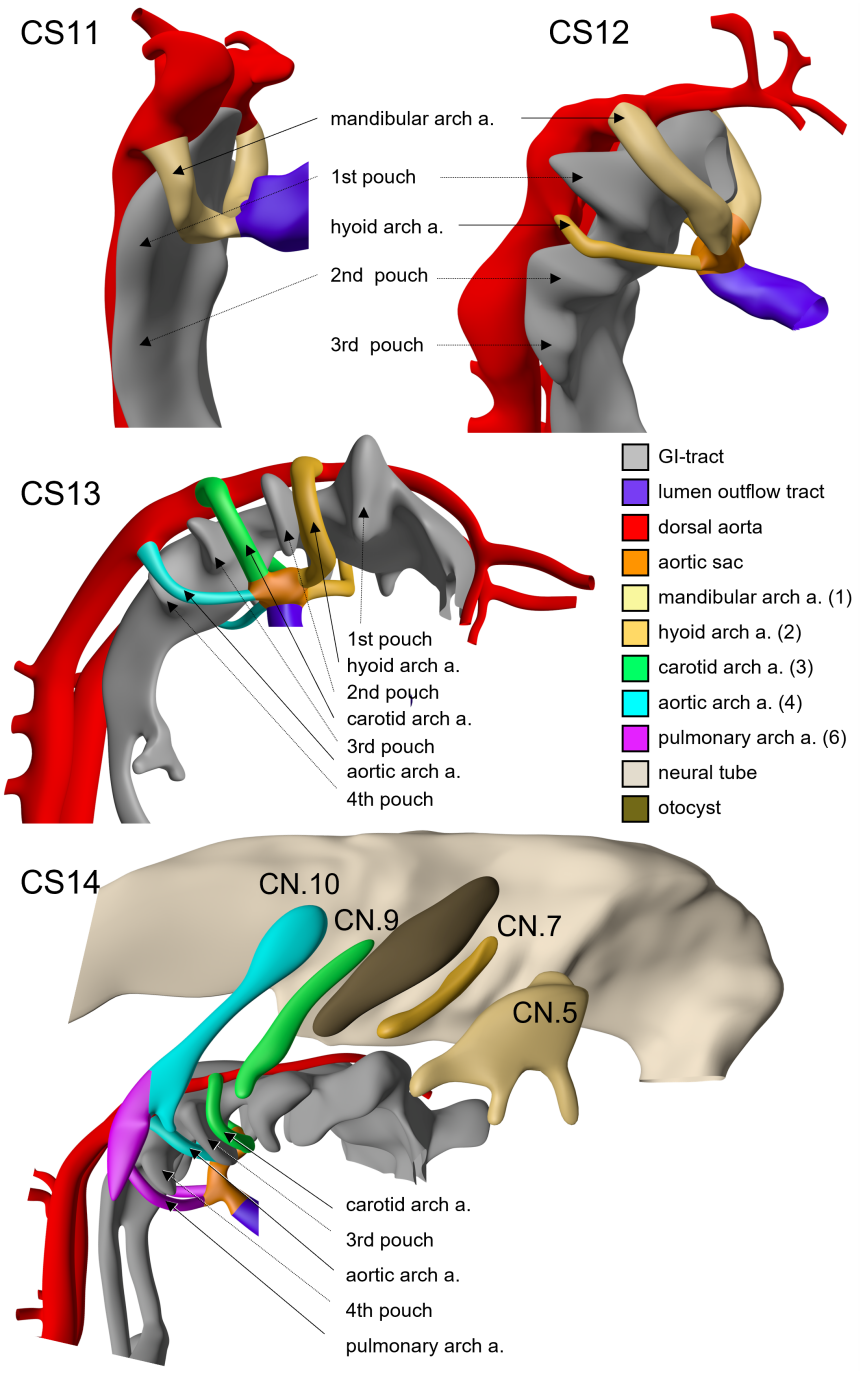
The images (right‐sided views) show reconstructions of the developing pharyngeal region of the human embryo between 29 and 34 days post‐fertilisation (CS11–CS14), revealing the appearance of the pharyngeal pouches, along with the arteries and nerves that extend through the arches delimited by the pouches.

The key initial event in the development of the pharyngeal arches is the formation of the pharyngeal pouches. It is the appearance of these entities that underpins the fundamental organisation of this region (Shone & Graham, 2014; Veitch et al., 1999). The pouches are outpocketings of the pharyngeal endoderm. They form at specific sites, protruding to contact the ectoderm. These points of apposition then define the pharyngeal pouches and the overlying ectodermal pharyngeal grooves or clefts (both terms, groove and cleft, are widely used and are interchangeable). In amniotes, four pharyngeal pouches form and these can be identified both morphologically and molecularly (Graham et al., 2019). In human development, the most cranial pouch forms first, together with the formation of the heart and the dorsal aortas, at Carnegie stage 9, ~26 days post conception (dpc). This configuration persists until Carnegie stage 11, (29 dpc) (Figure 1(a) and Figure S1). The second pouch can be recognised at Carnegie stage 12 (30 dpc) (Figure 1(b), Figure S2). The third and fourth pouches then form in turn. The third pouch can be recognised at Carnegie stage 13, (32 dpc) (Figure 1(c), Figure S3). The fourth pouch is identifiable from Carnegie stage 13 (Figure 1(d) and Figure S4). The additional embryonic populations contributing to the pharyngeal arches are constrained by this initial segmental organisation of the four pouches and the corresponding grooves/clefts. The neural crest cells emerge from the developing brain and migrate in three separate streams (Lumsden et al., 1991). The most cranial population arises from the posterior midbrain and anterior hindbrain. This fills the first pharyngeal arch. The central region of the hindbrain, centred on rhombomere 4, generates the neural crest cells which will fill the second pharyngeal arch. The most caudal stream is produced by the caudal hindbrain. It migrates between the otic vesicle and the most rostral somite. This is split by the positioning of the pouches and clefts and fills the three most caudal arches. The mesodermal population derives from its paraxial position, moving along with the neural crest cells to populate the arches.

The result of these processes is the generation of four pouches and five pharyngeal arches. The first, the most cranial arch, is bounded by the oral ectoderm cranially, and the apposition of the first pouch and groove/cleft caudally. The second arch is defined cranially by the first pouch and groove/cleft, having the second pouch and groove/cleft as its caudal border. The third and fourth arches are similarly confined by the pouches and grooves/clefts. The ultimate arch has an obvious cranial boundary formed by the fourth pouch and groove/cleft but has no clear caudal limit. Once the full complement of arches has been formed, the region undergoes major remodelling. The second, or hyoid, arch is markedly different from all the others. It expands greatly in size driven by localised proliferation at its caudal margin. This results in the second pharyngeal arch projecting caudally and forming a sinus between the second arch and the underlying tissue (Richardson et al., 2012). At this time, the more caudal arches are internalised, thereby losing their individual identities. Importantly, however, the internalisation of the caudal arches is not dependent on the expansion of the second arch (Poopalasundaram et al., 2023).

A key point arising from these considerations of development is the significant differences between the cranial and caudal arches. It is possible to assign definitive skeletal and connective tissue derivatives, along with muscles and endothelium, and sensory and motor innervation, to the cranial arches. This is not so for the caudal arches, which exist but transiently. The two most caudal arches do not generate any defined muscular and skeletal elements during the period of their existence. In current tables, these arches are generally assumed to form components of the larynx. Such derivatives, however, do not appear until long after the last two arches have been remodelled (de Bakker et al., 2017; Poopalasundaram et al., 2019). These two most caudal arches also differ from the more cranial arches, in that they do not have a single defined neurogenic placode/sensory ganglion associated with them. Rather, they form a common neurogenic placode, which generates the sensory neurons of the nodose ganglion, and they share their innervation from the tenth cranial, or vagus, nerve. The main derivatives of the two caudal arches are the blood vessels that form within them.

**FIGURE 2:**
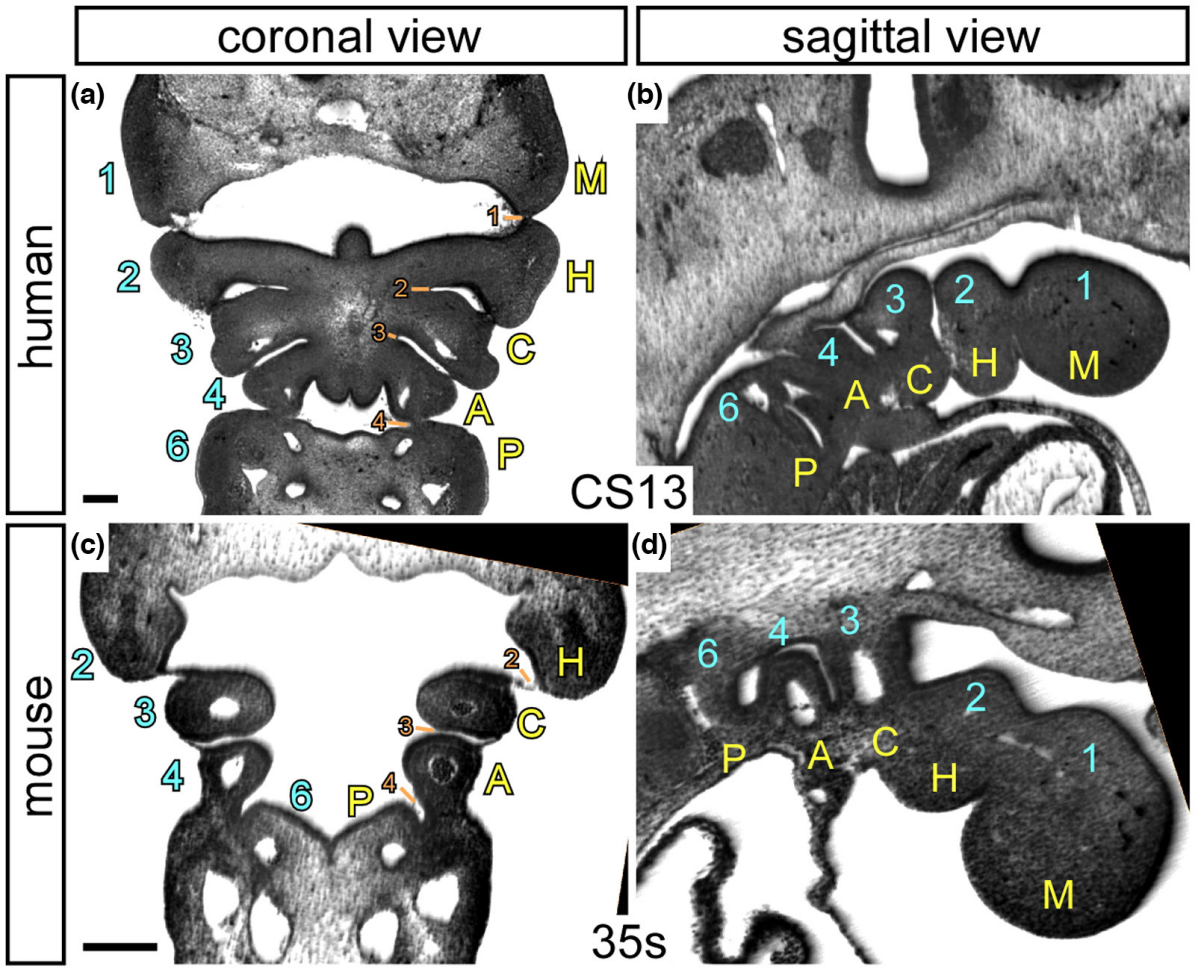
High‐resolution episcopic microscopy images of human (a, b) and mouse (c, d) embryos in transverse (a, c) and sagittal (b, d) views. The pharyngeal region is shown with the current labelling system indicated in blue numbers, with the proposed new labelling system in yellow letters (M, mandibular; H, hyoid; C, carotid; A, aortic; and P, pulmonary). Pharyngeal pouches are indicated with orange numbers. CS: Carnegie stage; s: somites. Scale, 200 μm.

Reconstructions of both human and murine embryos show unequivocally that there are but five arches formed during development (Figure 2). There is no transient additional ‘fifth’ arch in normal development. This means that the numbering of the arches needs to be revised. To provide clarity, we suggest that it would be best to move from a numerical approach to a descriptive one that employs clear labels. We propose that the most cranial arch be mandibular. The second arch can then be termed the hyoid, with the third being carotid, the fourth, the aortic, and the last one, the pulmonary arch. On this basis, in Table 1, we show each of the pharyngeal arches, with their revised terminology, emphasising the derivatives that can be ascribed to each arch in amniotes. A notable feature of this is that the main derivatives of two most caudal arches are the arteries that form within them. The pouches and grooves/clefts can still be numbered numerically running cranially to caudally (Figures 1 and 2). The first pouch and its groove/cleft will form the Eustachian tube and external auditory meatus. From the second pouch will be formed the palatine tonsils. The third pouch will give rise to the thymus and two of the parathyroid glands. The fourth pouch will form the remaining two parathyroid glands as well as the ultimobranchial body.

**TABLE 1:** The revised terminology for the pahryngeal arches and a list of the derivatives of each of the arches.

Arch		Derivative				
Old	New	Cartilage	Bone	Muscle	Nerve	Arch artery
1	Mandibular	Quadrate, Meckel's	Incus, Malleus	Tensor tympani, muscles of mastication, mylohyoid, anterior belly digastric, tensor veli palatini	Trigeminal, mandibular branch	First (transitory)
2	Hyoid	Reichert's	Stapes, styloid process of temporal bone, lesser horn, upper part of hyoid	Stapedius, stylohyoid, facial muscles	Facial	Second (transitory)
3	Carotid	‐	Greater horn and lower part of hyoid	Stylopharyngeus	Glosso‐pharyngeal	Carotid
4	Aortic	–	–	–	Vagus, pharyngeal branch	Aortic
Last	Pulmonary	–	–	–	Vagus, laryngeal branch	Pulmonary

## 5 SIGNIFICANCE TO TERMINOLOGY

**FIGURE 3:**
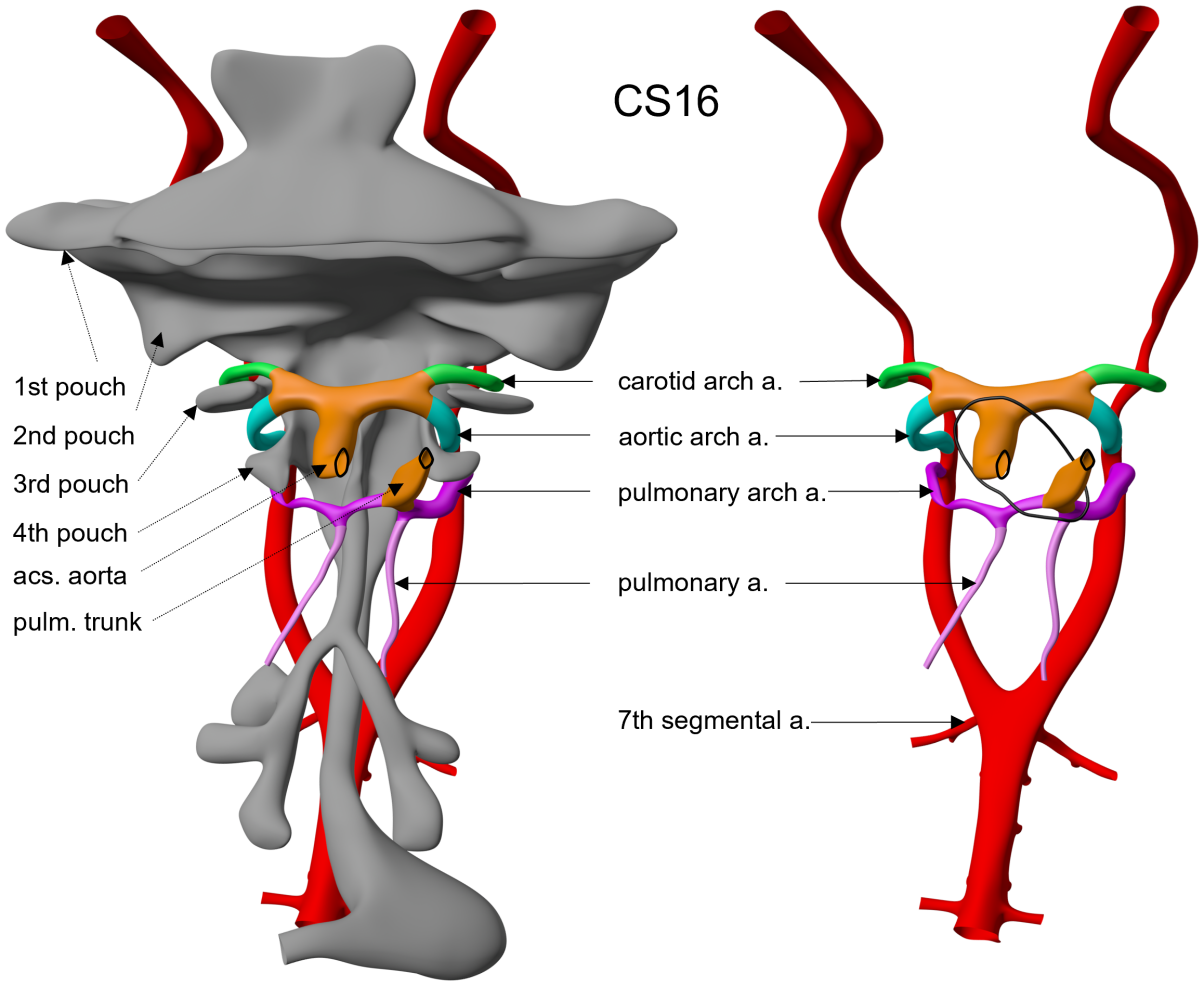
The images (ventral views) show a reconstruction of the developing pharyngeal region at Carnegie stage 16, which is the equivalent of around 39 days after fertilisation. The arch arteries remain relatively symmetrical at this stage but are remodelling to form the definitive systemic and pulmonary arterial channels. The arterial duct, previously usually described as the ‘left sixth arch artery’, is now better described as the left pulmonary arch artery. The derivatives of the aortic sac are displayed in orange and partially positioned intra‐ and extra‐pericardially. The black contour identifies the pericardial reflection (Figure modified after Hikspoors et al., 2022).

In terms of terminological change, our proposals will have most significance to those working in the field of congenital cardiac malformations. This is because it remains frequent to find congenital cardiac lesions interpreted on the basis of persistence of the postulated arteries of the fifth arch (Gerlis et al., 1987). Since we now know that there are never six arches formed during normal human development, it follows that there cannot be any putative ‘fifth arch arteries’. The entities previously interpreted on this basis can be shown to be the consequence either of persistent collateral channels or remodelling of the aortic sac (Anderson et al., 2020; Anderson & Bamforth, 2022). These facts make it the more important that a descriptive approach be adopted for ongoing interpretation, rather than taking an alternative approach and seeking simply to renumber the existing arches as one through five. It would be confusing for paediatric cardiologists should the arteries of the pulmonary arches themselves be described as ‘fifth arch arteries’. The main derivative of these arteries is the arterial duct, which is the persistent left pulmonary arch artery (Figure 3 and Figure S5).

## 6 CONCLUSION

We believe that the current understanding of the significance of the pharyngeal arches and the terminology used to discuss them is poor. We believe that it is important that this is updated. We suggest a move towards a nominal terminology. The current system of numbering from one to four and then six, causes confusion. This is particularly an issue in relationship to considerations of the pharyngeal arch arteries. As pointed out above, it would be equally confusing if the result of our review was to have the arch currently considered to be sixth to be renumbered as being fifth. We propose, therefore, a shift to a new nomenclature in which the arches are labelled nominally, from cranial to caudal—the first is the mandibular (M), the second the hyoid (H), the third the carotid (C), the fourth the aortic (A) and the last most caudal the pulmonary (P). The terms mandibular and hyoid, relate to the skeletal elements formed within these arches and are already routinely used for these structures. The new terminology for the three caudal arches aligns with that for the arch arteries, which are labelled correspondingly. The two most caudal arches do not form skeletal or muscular derivatives during the period of their existence and this labelling reflects the fact that these vessels are major derivatives of these arches.

## Supporting information


**Figure S1:** CS11 embryo at ~29 days after fertilisation (10.6084/m9.figshare.22140302). The first two pharyngeal pouches have become recognisable. The mandibular arch arteries have originated cranio‐ventral to the first pouch, connecting the heart with the dorsal aortae.

**Figure S2:** CS12 embryo at ~30 days after fertilisation (10.6084/m9.figshare.22140353). Note that the third pharyngeal pouch has become identifiable. The hyoid arch arteries are present in between the first and second pouch.

**Figure S3:** CS13 embryo at ~32 days after fertilisation (10.6084/m9.figshare.22140428). All four pouches are recognisable. While the mandibular arch arteries are not identifiable anymore, the carotid arch arteries have become apparent between the second and third pharyngeal pouches. The aortic arch arteries have originated at this stage as well, and are positioned in between the third and fourth pouches.

**Figure S4:** CS14 embryo at ~34 days after fertilisation (10.6084/m9.figshare.22140440). Neither the mandibular nor the hyoid arch arteries are recognisable anymore. Next to the carotid and aortic arch arteries, the pulmonary arch arteries have become apparent. Besides the developing arteries, this figure also shows the corresponding cranial nerves.

**Figure S5:** CS16 embryo at ~38 days after fertilisation (10.6084/m9.figshare.22140443). The arch arteries remain relatively symmetrical at this stage but are remodelling to form the definitive systemic and pulmonary arterial channels.

## Data Availability

Data sharing is not applicable to this article as no new data were created or analyzed in this study.
