# Supplementary figures and images for: A revised terminology for the pharyngeal arches and the arch arteries
Source: J Anat. 2023 May 29;243(4):564–9. doi: 10.1111/joa.13890 (PMC10485586; doi:10.1111/joa.13890)

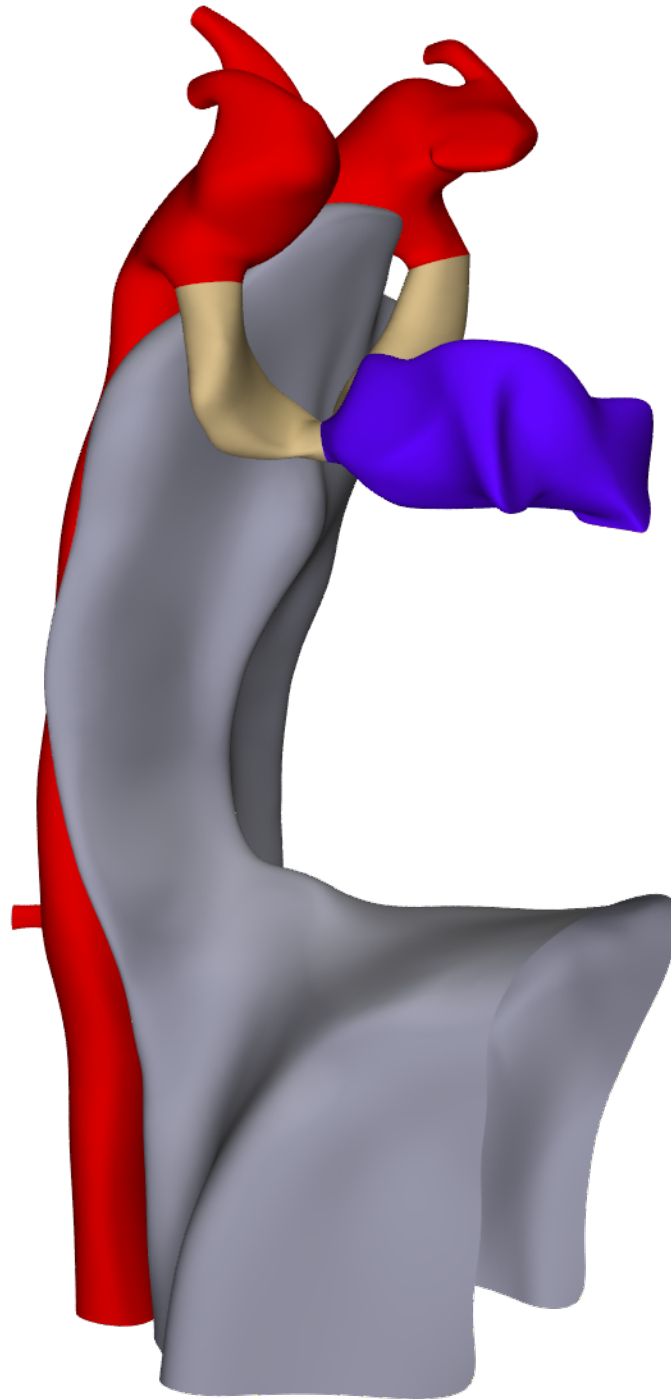

Supplement: Supplementary file 1 — Figure S1: CS11 embryo at ~29 days after fertilisation (10.6084/m9.figshare.22140302). The first two pharyngeal pouches have become recognisable. The mandibular arch arteries have originated cranio‐ventral to the first pouch, connecting the heart with the dorsal aortae. [file JOA-243-564-s003.pdf]

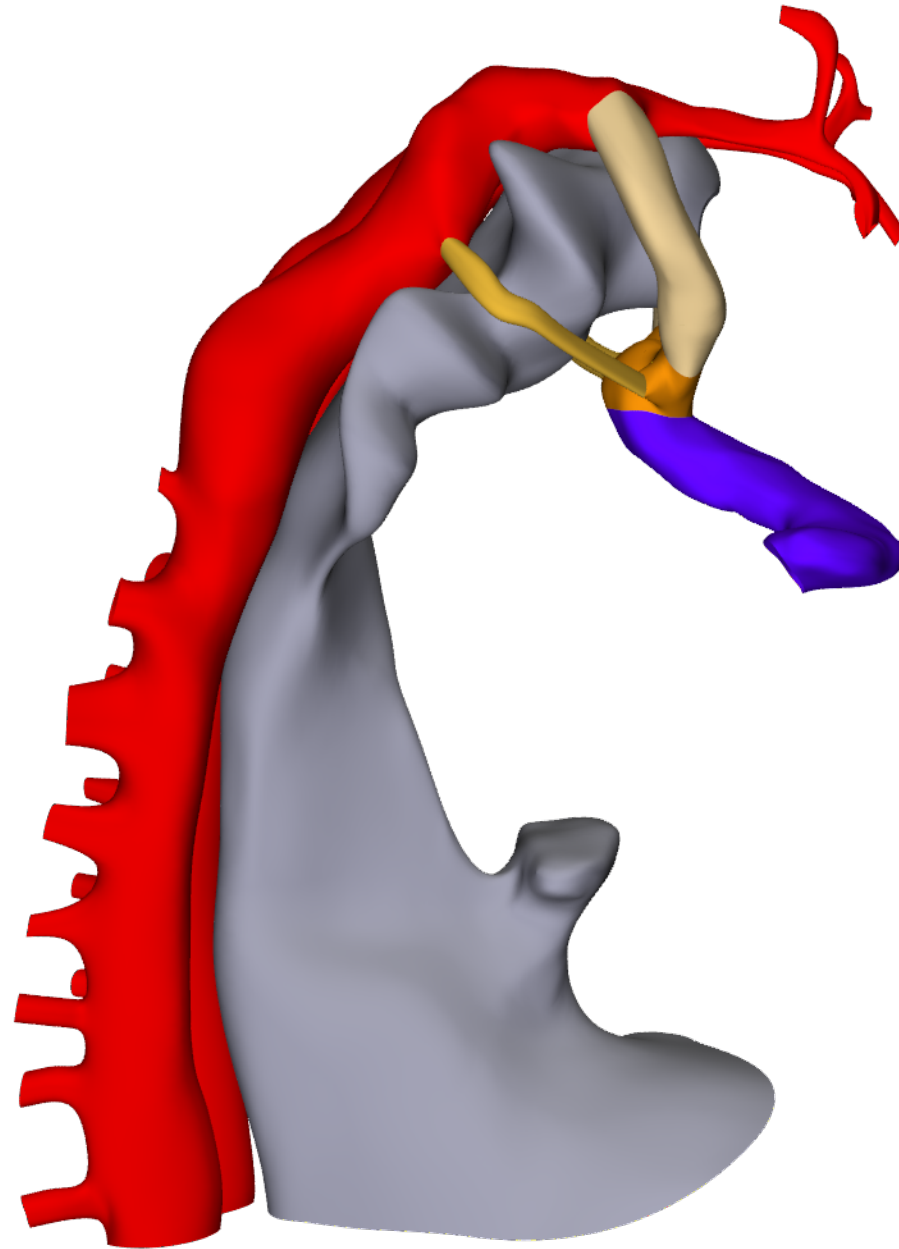

Supplement: Supplementary file 2 — Figure S2: CS12 embryo at ~30 days after fertilisation (10.6084/m9.figshare.22140353). Note that the third pharyngeal pouch has become identifiable. The hyoid arch arteries are present in between the first and second pouch. [file JOA-243-564-s002.pdf]

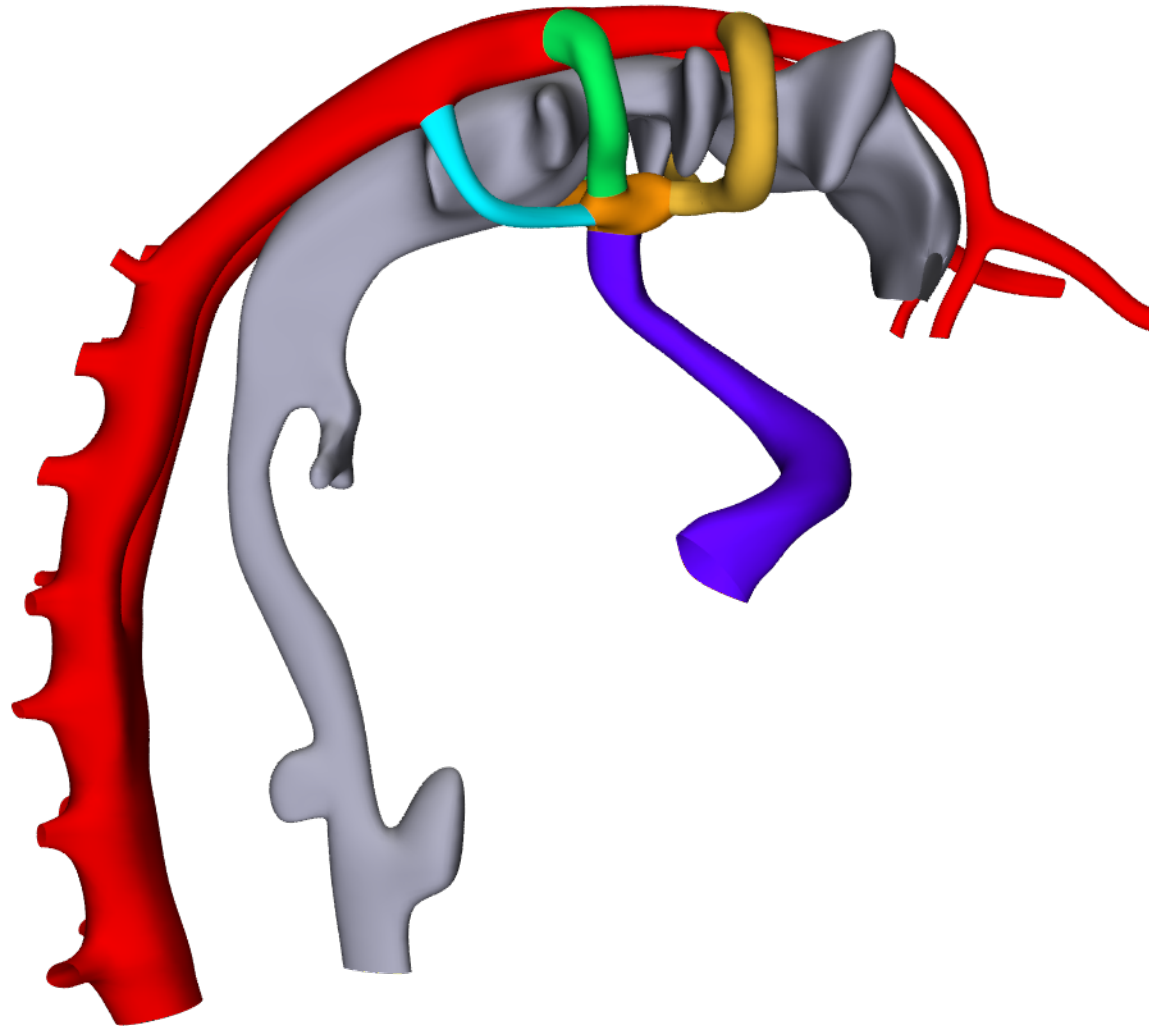

Supplement: Supplementary file 3 — Figure S3: CS13 embryo at ~32 days after fertilisation (10.6084/m9.figshare.22140428). All four pouches are recognisable. While the mandibular arch arteries are not identifiable anymore, the carotid arch arteries have become apparent between the second and third pharyngeal pouches. The aortic arch arteries have originated at this stage as well, and are positioned in between the third and fourth pouches. [file JOA-243-564-s001.pdf]

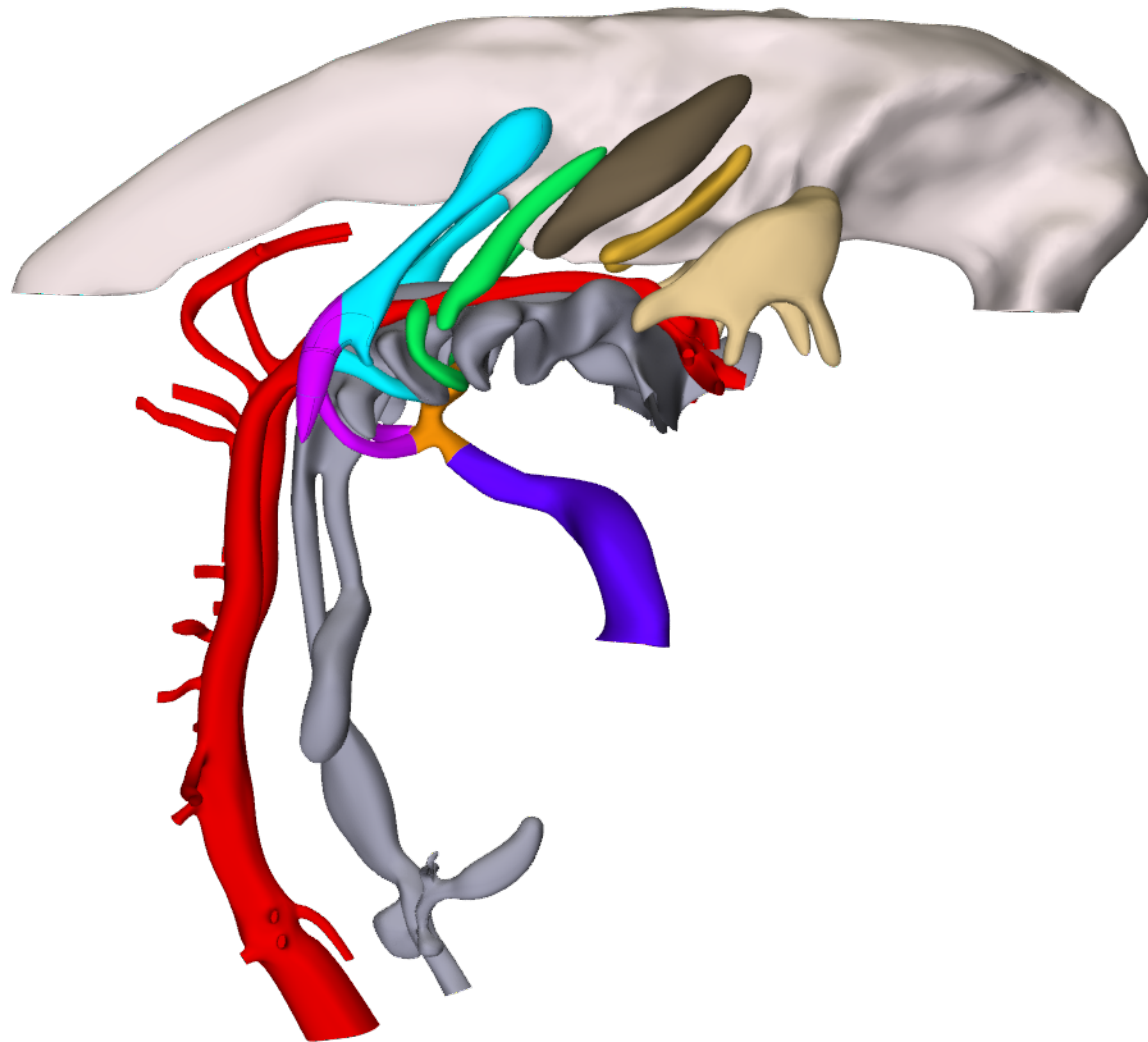

Supplement: Supplementary file 4 — Figure S4: CS14 embryo at ~34 days after fertilisation (10.6084/m9.figshare.22140440). Neither the mandibular nor the hyoid arch arteries are recognisable anymore. Next to the carotid and aortic arch arteries, the pulmonary arch arteries have become apparent. Besides the developing arteries, this figure also shows the corresponding cranial nerves. [file JOA-243-564-s005.pdf]

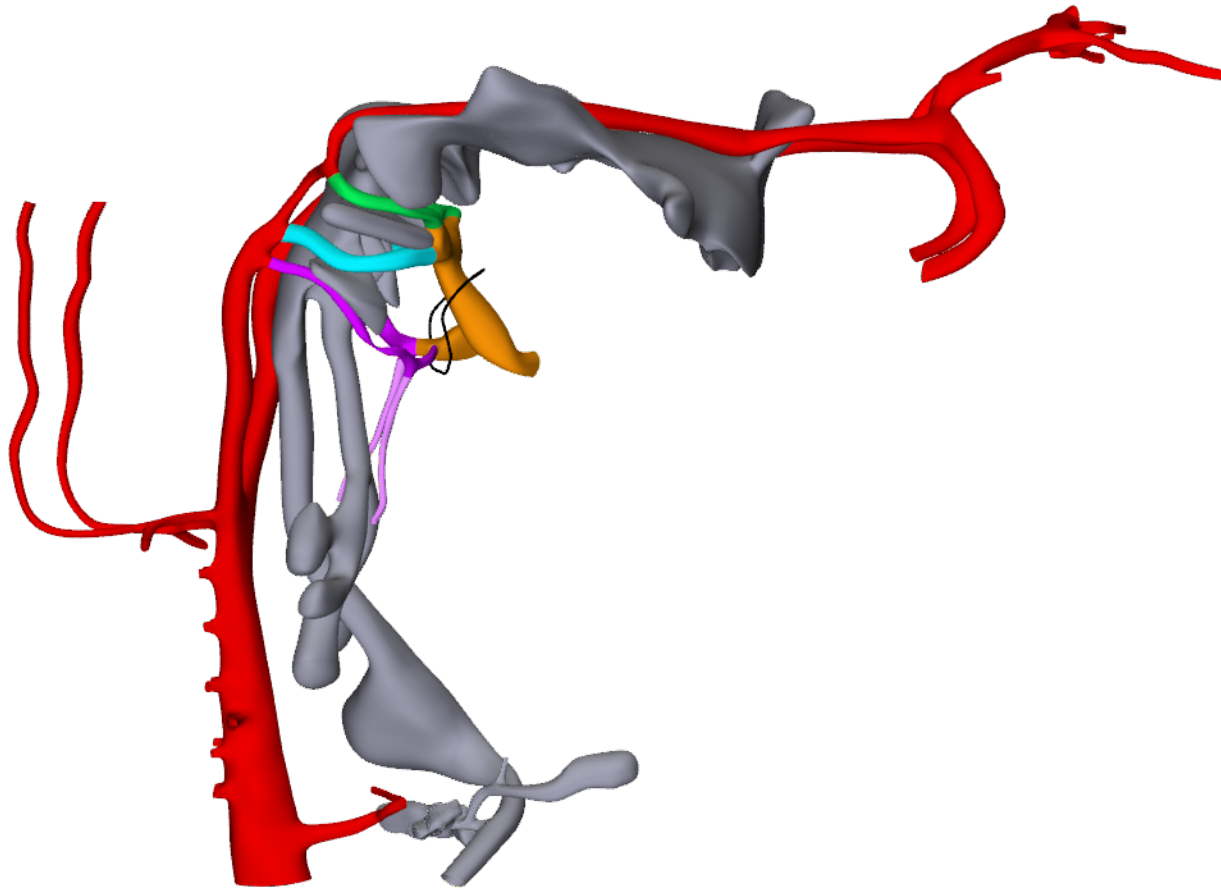

Supplement: Supplementary file 5 — Figure S5: CS16 embryo at ~38 days after fertilisation (10.6084/m9.figshare.22140443). The arch arteries remain relatively symmetrical at this stage but are remodelling to form the definitive systemic and pulmonary arterial channels. [file JOA-243-564-s004.pdf]
